# Intraoperative Real-Time Near-Infrared Image-Guided Surgery to Identify Intracranial Meningiomas *via* Microscope

**DOI:** 10.3389/fnins.2022.837349

**Published:** 2022-05-04

**Authors:** Jun Muto, Yutaka Mine, Yuya Nishiyama, Kazuhiro Murayama, Seiji Yamada, Daijiro Kojima, Motoharu Hayakawa, Kazuhide Adachi, Mitsuhiro Hasegawa, John Y. K. Lee, Yuichi Hirose

**Affiliations:** ^1^Department of Neurosurgery, Fujita Health University, Toyoake, Japan; ^2^Department of Neurosurgery, Saiseikai Yokohamashi Tobu Hospital, Yokohama, Japan; ^3^Department of Radiology, Fujita Health University, Toyoake, Japan; ^4^Department of Pathology, Fujita Health University, Toyoake, Japan; ^5^Department of Neurosurgery, University of Pennsylvania, Philadelphia, PA, United States

**Keywords:** enhanced permeability retention, intracranial meningioma, metabolic navigation, near-infrared, second window indocyanine green, fluorescence

## Abstract

Meningiomas are a common pathology in the central nervous system requiring complete surgical resection. However, in cases of recurrence and post-irradiation, accurate identification of tumor remnants and a dural tail under bright light remains challenging. We aimed to perform real-time intraoperative visualization of the meningioma and dural tail using a delayed-window indocyanine green (ICG) technique with microscopy. Fifteen patients with intracranial meningioma received 0.5 mg/kg ICG a few hours before observation during the surgery. We used near-infrared (NIR) fluorescence to identify the tumor location. NIR fluorescence could visualize meningiomas in 12 out of 15 cases. Near-infrared visualization during the surgery ranged from 1 to 4 h after the administration of ICG. The mean signal-to-background ratio (SBR) of the intracranial meningioma in delayed-window ICG (DWIG) was 3.3 ± 2.6. The ratio of gadolinium-enhanced T1 tumor signal to the brain (T1BR) (2.5 ± 0.9) was significantly correlated with the tumor SBR (*p* = 0.016). K*^trans^*, indicating blood–brain barrier permeability, was significantly correlated with tumor SBR (*p* < 0.0001) and T1BR (*p* = 0.013) on dynamic contrast-enhanced magnetic resonance imaging (MRI). DWIG demonstrated a sensitivity of 94%, specificity of 38%, positive predictive value (PPV) of 76%, and negative predictive value (NPV) of 75% for meningiomas. This is the first pilot study in which DWIG fluorescence-guided surgery was used to visualize meningioma and dural tail intraoperatively with microscopy. DWIG is comparable with second-window ICG in terms of mean SBR. Gadolinium-enhanced T1 tumor signal may predict NIR fluorescence of the intracranial meningioma. Blood–brain barrier permeability as shown by K*^trans^* on dynamic contrast-enhanced MRI can contribute to gadolinium enhancement on MRI and to ICG retention and tumor fluorescence by NIR.

## Introduction

Meningiomas are the most frequently occurring intracranial tumors in adults, accounting for one third of all cases. The gold standard treatment for symptomatic meningioma is resection (Mirimanoff et al., 1985), but even after removal and maximal adjunctive radiation therapy, the median time to radiological progression is 12, 7, and 2 years for World Health Organization (WHO) grades I, II, and III meningiomas, respectively (Olar et al., 2015).

Currently, the differentiation between the dural or adjacent bone invasion and reactive tissue in meningioma seems to be limited. Chances of safe gross total resection without neurological defects may decrease due to the tumor’s location, invasion of the dura and/or bone, and involvement of neurovascular structures. Fluorescence-guided surgery may be a helpful tool in evaluating the extent of resection, especially in patients with complex meningiomas. 5-Aminolevulinic acid (5-ALA), a prodrug in the porphyrin family, has been shown to facilitate tumor identification, improve tumor resection rates in meningioma (Dijkstra et al., 2019; Valdes et al., 2019), and prolong overall survival in glioblastoma (Stummer et al., 2000, 2006). However, 5-ALA has limitations, i.e., fluorescence in the visible light spectrum (400–700 nm) is highly absorbed by endogenous fluorophores, a lack of tissue penetrance, and confounding with background brain autofluorescence (Stummer et al., 2006).

Fluorescence-guided neurosurgery is useful, especially for recurrent and radiated cases, where it is difficult to identify the exact tumor location by direct observation under a bright light. Indocyanine green (ICG) has been used in neurosurgery as a vascular contrast agent in the evaluation of aneurysms by facilitating the assessment of arterial and venous flow (Hide et al., 2015). Intraoperative video angiography shows real-time blood flow during surgery with a bolus of 5–25 mg per operation (Ueba et al., 2013). Recently, second-window ICG (SWIG) for tumor lesions was reported in which a high dose of 5 mg/kg of ICG was administered to a patient harboring a brain tumor up to 24 h before surgery, and near-infrared (NIR) fluorescence from the tumor itself could be observed during the surgery. The usefulness of SWIG has been shown in meningioma (Lee et al., 2017a), glioblastoma (Lee et al., 2016), and metastatic tumors (Lee et al., 2017b; Muto et al., 2021).

However, SWIG requires a dose administration of ICG corresponding to the maximum safe capacity for biological use, which may cause side effects and limit its application to patients and require administration 1 day before observation. We modified the dose and timing of administration to 0.5 mg/kg ICG during the surgery, and NIR fluorescence can be observed more than 1 h after administration. We defined this modified technique as “delayed window ICG (DWIG).” The reason for the observation time was that Zeh et al. (2017) found that NIR fluorescence in glioblastomas in a mouse model peaked at 1 h post-infusion and plateaued 24 h post-infusion. Currently, 0.5 mg/kg of ICG administered intraoperatively has been used to evaluate real-time blood flow in neurosurgery with a few side effects. Using the DWIG technique, fluorescence from the tumor can be detected more than 1 h after intraoperative administration of 25 mg of ICG. Considering these findings, we hypothesized that (1) the administration of ICG more than 1 h before surgery might be feasible for visualizing the tumor and dural tail compared with the normal brain parenchyma, (2) there is a correlation between gadolinium-enhanced magnetic resonance imaging (Gd-MRI) and ICG fluorescence of the lesions, and (3) the mechanism of ICG fluorescence is implicated by vascular permeability.

We aimed to use DWIG after administering 0.5 mg/kg of ICG intraoperatively with delayed observation for intracranial meningioma and the dural tail, evaluate its efficacy, and clarify its mechanism in this pilot study.

## Materials and Methods

### Study Design and Ethical Approval

This prospective study was approved by the Fujita Health University Clinical Research Ethical Committee (CRB4180003) and Saiseikai Yokohamashi Tobu Hospital (20200137). Informed consent was obtained from all patients.

### Patient Population

Fifteen meningiomas in 15 patients were enrolled in this study. All patients underwent Gd-MRI, computed tomography scan, and surgical resection with intravenous ICG administration.

### Near-Infrared Imaging System

The KINEVO microscope (KINEVO 900, Carl Zeiss Meditec AG, Jena Germany) was used for the 15 patients, with the FLOW 800 application that utilized an NIR excitation light source (805 nm) and camera (820–860 nm). NIR images were captured and stored for further evaluation.

### Surgery and Delayed-Window Indocyanine Green Technique

Each patient received intravenous ICG (0.5 mg/kg) (C43H47N2NaO6S2, Daiichi Sankyo, Tokyo, Japan) mixed with 10 ml of 0.9% saline during the craniotomy at the beginning of the surgery. NIR visualization ranged from 1 to 4 h after the administration of ICG. After exposing the dura mater, the microscope was introduced, the operating room was dimmed, and light in the NIR wavelength was irradiated from the illumination system. The NIR fluorescence was sometimes detected over the dura mater in cases where the tumor was attached to the convex side of the dura. Then the dura mater was incised, and the intradural tumor was exposed. Subsequently, NIR fluorescence was detected from the tumor itself using an illumination system. Fluorescence excitation and exposure were registered when the tumor was exposed for the first time. At that point, the tumor seemed to be most visible compared with the normal brain parenchyma, and fluorescence excitation and imaging settings were sustained throughout the remainder of the surgery. The tumors and dural tail were dissected and totally removed under DWIG based on the fluorescence guidance.

### Dynamic Contrast-Enhanced Magnetic Resonance Imaging Examination and Post-processing

**FIGURE 1:**
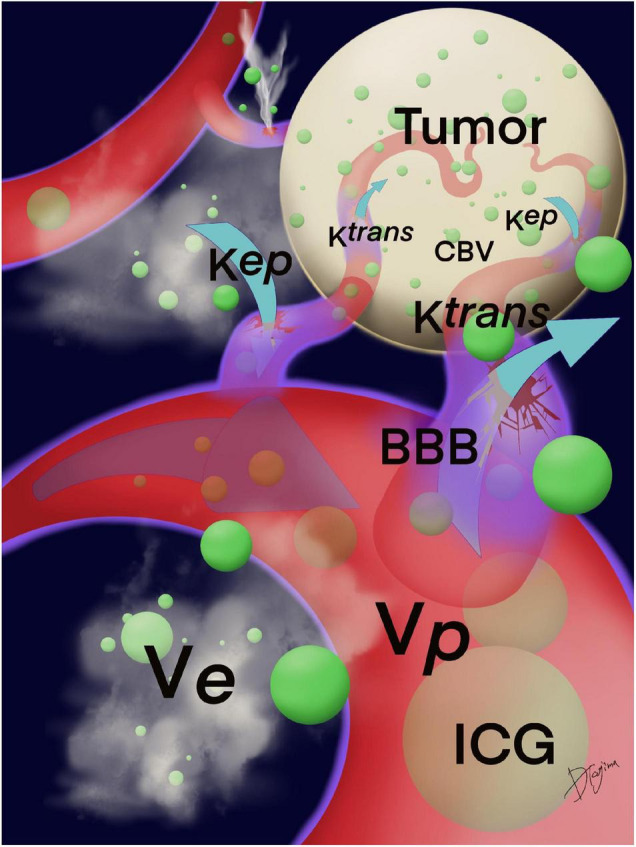
Schema of K*^trans^*, K*^ep^*, V*^e^*, and V*^p^*. K*^trans^* indicates the permeability from the intravascular to the extravascular space; K*^ep^*, from the extravascular to the intravascular space; V*^e^*, the volume of the extravascular extracellular space; and V*^p^*, the fractional plasma volume. The BBB around the tumor was damaged, and ICG may leak from vascular structures at the compromised BBB that were calculated by the permeability. Green circles are ICG particles. BBB, blood–brain barrier; ICG, indocyanine green.

Magnetic resonance imaging (MRI) examinations were performed using a 3T MRI system (Vantage Titan 3T; Canon Medical Systems Corporation, Otawara, Tochigi, Japan) with a 32-channel head coil. Axial dynamic contrast-enhanced (DCE) MRI was performed after intravenous administration of a contrast agent using a 3D fast field echo quick sequence that provided coverage of the whole brain tumor using the following parameters: matrix size, zero-filling matrix 512 × 512 (acquisition matrix 186 × 256); field of view, 220 mm × 220 mm; repetition time, 5.5 ms; echo time, 2.5 ms; flip angle, 15°; and section thickness, 5 mm. Thirty-one dynamic consecutive volumes, each including 21 sections to cover the tumor based on T2-weighted images, were obtained every 10 s, giving a total measurement time of 5 min and 4 s. The contrast agent gadobutrol (Gadovist; Bayer, Osaka, Japan, 0.05 ml/kg body weight) was injected intravenously as a bolus through a driven autoinjector (Sonic Shot GX; Nemoto, Japan) at a rate of 1 ml/s, followed by an intravenous bolus injection of 30 ml of physiological saline solution at 1 ml/s. Post-processing of DCE perfusion MR images was performed using dedicated post-processing software (Olea Sphere V3.0, Olea Medical, Vitrea Workstation V7.1, Canon Medical Systems Corporation). The pharmacokinetic model introduced by Tofts et al. can also be used to calculate the volume transfer constant K*^trans^* (/min) from the intravascular to the extravascular space, K*^ep^* (= K^*trans*/^V*^e^*)(/min)from the extravascular to the intravascular space, the volume of the extravascular extracellular space V*^e^* (ml/100 ml of tissue), and fractional plasma volume V*^p^* (ml/100 ml of tissue) (Tofts and Kermode, 1991; Tofts et al., 1999). The two-compartment pharmacokinetic model proposed by the extended model (Tofts et al., 1999) asserts that blood vessels and tissues are regarded as independent compartments. Each parameter is calculated from the relationship between contrast media concentrations at the target and reference sites based on this two-compartment model with the following parameters: the rate constant for tissue migration of contrast media (K*^trans^*), the volume ratio of the extracellular space outside the vessel (V*^e^*), and the volume ratio of the intravascular component (V*^p^*). Based on this two-compartment pharmacokinetic model for DCE-MRI, we used the perfusion analysis method to calculate the permeability parameter (Tofts and Kermode, 1991) as only K*^trans^*. A schematic of K*^trans^*, K*^ep^*, V*^e^*, and V*^p^* is depicted in Figure 1.

### Image Analysis and Statistical Analysis

In all cases, DCE-MRI and ICG-NIR videos were stored to assess the feasibility and efficacy of real-time ICG-NIR imaging for intracranial meningioma and analyze the correlation between DCE-MRI and ICG-NIR. We obtained a background reading from the adjacent normal brain to generate a signal-to-background ratio (SBR). Additionally, region of interest (ROI) analysis was performed to quantify the amount of fluorescence from the tissues. We selected five ROIs corresponding to the tumor lesions and normal tissue lesions on the images and analyzed their average values. We drew five ROIs on Gd-enhanced T1 MRI to compare the enhanced tumor with the normal brain parenchyma and analyzed their average values, which we called the T1-Gd enhancement to normal brain ratio (T1BR) using ImageJ software (NIH Image, Bethesda, MD, United States). Univariate analyses were conducted using the chi-square or Fisher’s exact test to compare categorical variables and the unpaired t-test or Mann–Whitney rank-sum test and simple linear regression analysis for continuous variables. Statistical significance was set at *p* < 0.05. Statistical analyses were performed using JMP 14.1.0 (SAS Institute Inc., Cary, NC, United States).

### Pathological Evaluation

Frozen tumor sections were prepared in a standard fashion. We collected samples of the dura mater and tumor, and numbered each sample during surgery. Histopathological analyses were performed, and tissue samples were examined and cataloged. Permanent sections were chemically fixed and embedded in paraffin blocks at a 5 μm thickness. Vertical sectioning was performed along with hematoxylin and eosin staining. Each tissue sample was assessed for the presence of tumor-specific cells by certified pathologists and correlated with fluorescence during surgery by neurosurgeons separately.

### Representative Cases

#### Case 1 (Patient No. 5)

**FIGURE 2:**
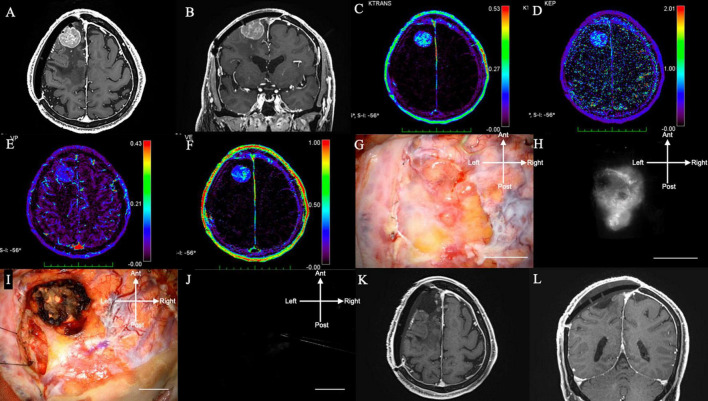
(Case 5). Convexity meningioma in the right frontal lobe displaying NIR fluorescence using a KINEVO microscope. **(A)** Axial and **(B)** sagittal Gd-enhanced T1-weighted pre-operative MRI showing the ring enhanced lesion in the left frontal lobe. **(C)** K*^trans^*, **(D)** K*^ep^*, **(E)** V*^e^*, and **(F)** V*^p^* on additional MRI. **(G)** Bright light image of the dura mater. **(H)** NIR fluorescence of the dura mater showing NIR fluorescence localized to the tumor. **(I)** Bright light image. **(J)** No NIR fluorescence after resection. **(K)** Axial and **(L)** sagittal Gd-enhanced T1-weighted postoperative MRI showing no residual lesion. NIR, near-infrared; Gd, gadolinium; MRI, magnetic resonance imaging; K*^trans^*, volume transfer constant; K*^ep^*, rate constant; V*^e^*, volume of the extravascular extracellular space; V*^p^*, vascular plasma volume.

An 80-years-old woman, who had undergone two surgical removals and two gamma knife radiations, presented with seizures and consulted with our department. The tumor was pathologically diagnosed as an anaplastic meningioma. Preoperative Gd-MRI revealed convexity meningioma recurrence (axial in Figure 2A and coronal in Figure 2B), which showed moderate intensity on K*^trans^* (Figure 2C), low intensity on K*^ep^* (Figure 2D), low intensity on V*^p^* (Figure 2E), and moderate intensity on V*^e^* (Figure 2F) on DCE-MRI. She received 0.5 mg/kg of ICG 1 h before the observation during surgery. During surgery, the tumor could be seen at the dura mater with granulation (Figure 2G). NIR fluorescence could be detected in the dural view and was confirmed by NIR before the dura incision (Figure 2H). In recurrent cases, it is difficult to distinguish tumor recurrence from normal dura mater with granulation under bright light. Following tumor resection with a margin, there was no tumor remnant under bright light in this case (Figure 2I) and no NIR fluorescence at the end of surgery, which confirmed that the tumor had been completely removed (Figure 2J). NIR fluorescence helped surgeons find the lesions that were difficult to identify under bright light, especially in recurrent and radiated cases. Post-operative Gd-MRI showed gross total resection (Figures 2K,L). There was no recurrence of symptoms for 8 months (Supplementary Video 1).

#### Case 2 (Patient No. 14)

**FIGURE 3:**
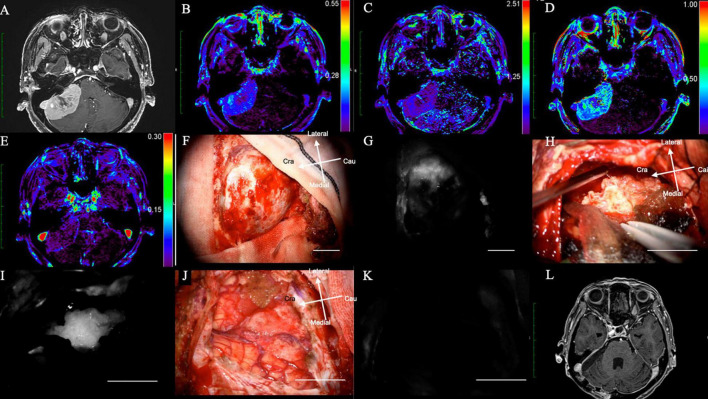
(Case 14). Meningioma at the right cerebellopontine angle displaying NIR fluorescence by microscope using a KINEVO microscope. **(A)** Axial Gd-enhanced T1-weighted preoperative MRI showing the ring enhanced lesion in the right frontal lobe; **(B)** K*^trans^*, **(C)** K*^ep^*, **(D)** V*^e^*, and **(E)** V*^p^* on additional MRI. **(F)** Bright light image of the dura mater. **(G)** NIR fluorescence of the dura mater. **(H)** Tumor location during resection under bright light. **(I)** NIR fluorescence of the dura mater showing the exact tumor location with brain shift during tumor resection. **(J)** Bright light image. **(K)** No NIR fluorescence after tumor resection showing total removal of the tumor. **(L)** Axial Gd-enhanced T1-weighted postoperative MRI showing no residual lesion. NIR, near-infrared; Gd, gadolinium; MRI, magnetic resonance imaging; K*^trans^*, volume transfer constant; K*^ep^*, rate constant; V*^e^*, volume of the extravascular extracellular space; V*^p^*, vascular plasma volume.

A 72-years-old woman presented with hearing disturbance and gait instability and was referred to our hospital. Her Gd-MRI revealed an enhanced mass (35 mm in maximum diameter) at the right cerebellum, suggesting meningioma (Figure 3A). It showed moderate intensity on K*^trans^* (Figure 3B), low intensity on K*^ep^* (Figure 3C), moderate intensity on V*^p^* (Figure 3D), and low intensity on V*^e^* (Figure 3E) on DCE-MRI.

The intracranial tumor was surgically treated with neuronavigation and NIR in the same manner as in Case 1. After the craniotomy, the dura mater was exposed (Figure 3F), and NIR fluorescence detected the tumor at the dural view, which helped determine the dural incision (Figure 3G). During tumor removal, the surgeon could identify the tumor under bright light (Figure 3H) and confirm its exact location during resection: in addition, residual tumor could be identified using NIR fluorescence.

Residual tumor could be identified, as well as that of the residual tumor, using NIR (Figure 3I).

After tumor removal, no residual tumor was observed under bright light (Figure 3J) and NIR (Figure 3K). Post-operative Gd-MRI showed no enhanced mass (Figure 3L). The patient’s symptoms improved dramatically after surgery, and she was discharged from the hospital without any neurological deficits (Supplementary Video 2).

## Results

### Delayed-Window Indocyanine Green Shows Near-Infrared Fluorescence of Intracranial Meningioma *via* Microscope

Fifteen patients were enrolled in this study. Eleven patients were female, and their mean age was 60 years (range 28–81 years). In total, 15 meningiomas were treated (total removal: 12; partial removal: 3). Patient characteristics are summarized in Table 1. Two representative cases are shown in Figure 2 (convexity) and Figure 3 (cerebellopontine angle). As mentioned previously, in this study, we modified the SWIG technique, in which a high dose of 5 mg/kg of ICG is administered up to 24 h before surgery to a patient harboring a brain tumor, to DWIG, in which 0.5 mg/kg of ICG is administered during surgery and observation of fluorescence occurs more than 1 h after administration.

**TABLE 1:** Data of 15 patients with intracranial meningioma, including age, gender, tumor location, pathology, tumor grade, MIB-1(%), time from ICG injection (hours), resection rate.

	Age	Gender	Location	Pathology	Tumor grade	MIB-1(%)	Observation time (hours)	Resection
1	57	M	Convexity	Angiomatous	1	9.5	1	GTR
2	28	F	Convexity	Meningothelial	1	4.5	3	PR*1
3	64	F	Sphenoid Ridge	Secretory	1	2.7	2	GTR
4	81	F	Sphenoid Ridge	Meningothelial	1	1.5	3	GTR
5	80	M	Convexity	Anaplastic	3	37.5	1	GTR
6	53	F	Sphenoid Ridge	Fibrous	1	1	2	GTR
7	76	F	Falx	Secretory	1	1.7	2	GTR
8	54	M	Plunum sphenoidale	Fibrous	1	2.4	2	GTR
9	55	M	Petroclival	Transitional	1	2.3	4	PR(*1)
10	62	F	Petroclival	Transitional	1	4.5	2	PR(*1)
11	54	F	Convexity	Angiomatous	1	2.7	1	GTR
12	36	F	Sphenoid Ridge	Meningothelial	1	5	2	GTR
13	53	F	Petroclival	Meningothelial	1	0.8	1	PR(*1)
14	72	F	CP Angle	Fibrous	1	2.3	3	GTR
15	77	F	Convexity	Atypical	2	8.2	2	GTR

*ICG, indocyanine green; M, male; F, female; GTR, gross tumor resection; PR, partial resection.*

Delayed-window ICG could detect and determine the location of meningiomas by NIR fluorescence. The average time from ICG infusion to observation was 2.1 ± 0.2 h (Table 1). Twelve out of 15 (80%) patients showed stronger NIR fluorescence in the tumor than in the surrounding brain parenchyma. The SBR from the tumor itself was 3.3 ± 2.6 (Table 2).

**TABLE 2:** Data of 15 patients with intracranial meningioma, including fluorescence, NIR tumor SBR, ratio of Gd-enhanced T1 tumor to normal brain, signal intensity of fluorescence in NIR and the scores of K^trans^, K^ep^, V^e^, and V^p^ on MRI.

Case	Fluorescence	SBR	T1BR	Signal visible with near-infrared camera	K*^trans^*	K*^ep^*	V*^e^*	V*^p^*
1	No	0.7	2.4	Weak	0.1	1.2	0.01	0.63
2	Positive	1.9	1.8	Moderate	0.16	0.62	0.28	0.25
3	No	0.8	1.9	Weak	0.9	1.7	0.52	0.24
4	Positive	1.8	2.7	Moderate	0.11	0.040	0.45	0.42
5	Positive	3.2	1.9	Strong	0.15	0.53	0.28	0.08
6	Positive	2.3	2.9	Strong	0.18	0.3	0.05	0.81
7	Positive	8.6	3.9	Strong	0.4	0.71	0.57	0.11
8	Positive	2.0	2.6	Moderate	0.21	2.02	0.97	0.03
9	Positive	2.7	2.9	Strong	0.24	0.71	0.95	0.24
10	Positive	3.7	1.7	Strong	0.22	0.46	0.46	0.49
11	No	0.006	2.1	Weak	0.06	0.07	0.08	0.47
12	Positive	7.0	2.5	Strong	0.36	0.51	0.15	0.2
13	Positive	7.5	5.3	Strong	0.52	0.59	0.88	0.46
14	Positive	4.0	1.6	Strong	0.13	0.40	0.36	0.06
15	Positive	3.6	1.9	Strong	0.3	1.7	0.48	0.1

*NIR, near-infrared; SBR, signal-to-background ratio; Gd, gadolinium; K^trans^, volume transfer constant; K^ep^, rate constant; V^e^, volume of the extravascular extracellular space; V^p^, vascular plasma volume; MRI, magnetic resonance imaging; T1BR, ratio of gadolinium-enhanced T1 tumor signal to the brain.*

In cases with NIR-positive meningiomas, no residual tumor was observed after surgery by NIR. Moreover, post-operative MRI did not show any residual lesions, implying that ICG-NIR can detect residual tumor during surgery. The SBR was not significantly correlated with some variables inherent to the tumor such as Ki-67 (*p* = 0.79), tumor size (*p* = 0.42), and maximum diameter (*p* = 0.40) (data not shown). Blood tests and physical and neurological examinations did not reveal any side effects for 3 months after the administration of ICG.

### T1-Gd Enhancement to Normal Brain Ratio on Magnetic Resonance Imaging

**FIGURE 4:**
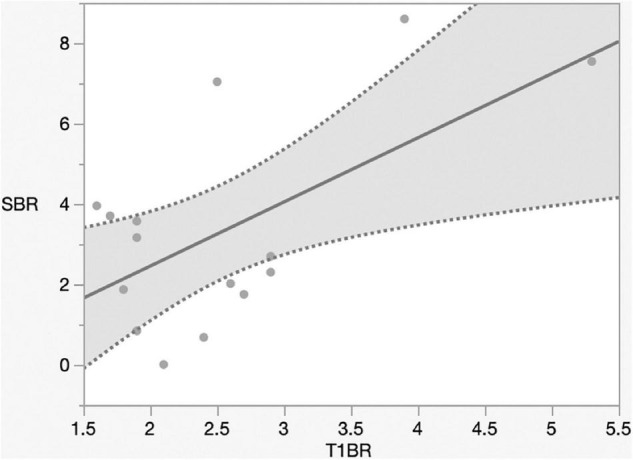
Linear regression plot of NIR. SBR of the tumor versus magnetic resonance imaging signal intensity ratio of the tumor on T1-contrast scan. The SBR increased with the ratio of gadolinium-enhanced T1 tumor signal to the normal brain (*p* = 0.016, R^2^ = 0.36). NIR, near-infrared; SBR, signal–background ratio; K*^trans^*, volume transfer constant; T1BR, ratio of gadolinium-enhanced T1 tumor signal to the brain.

Preoperative Gd enhancement may predict NIR fluorescence (Lee et al., 2016). Next, we assessed the correlation between Gd-MRI and NIR fluorescence. The mean T1-Gd enhancement to normal brain ratio (T1BR) was 2.5 ± 0.9. We evaluated the relationship between T1BR and SBR using linear regression analysis. T1BR was significantly correlated with the SBR of NIR fluorescence (*p* = 0.016, R^2^ = 0.36) as shown in Figure 4 but not with Ki-67 (*p* = 0.28), tumor size (*p* = 0.47), and diameter (*p* = 0.27) (data not shown).

### Blood–Brain Barrier Permeability May Cause Near-Infrared Fluorescence

**FIGURE 5:**
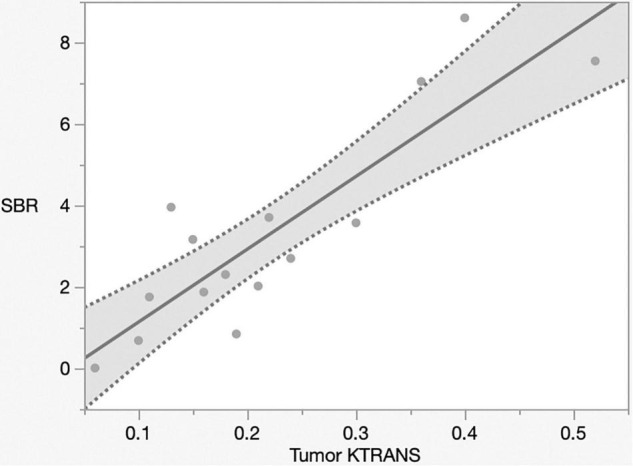
Linear regression plot of NIR; SBR of tumor from the brain surface versus K*^trans^*. The SBR increased with K*^trans^* on magnetic resonance imaging (*p* < 0.0001, R^2^ = 0.77). NIR, near-infrared; SBR, signal–background ratio; K*^trans^*, volume transfer constant.

**FIGURE 6:**
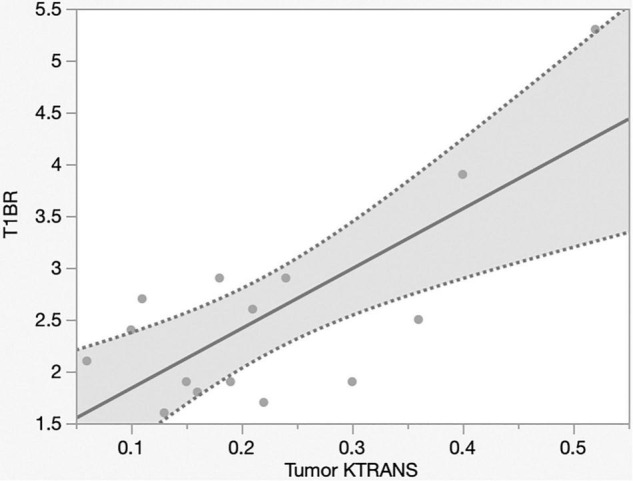
Linear regression plot of NIR; MRI signal intensity ratio of the tumor on T1-contrast scan versus K*^trans^* on MRI. T1BR increased with K*^trans^* (*p* = 0.013, R^2^ = 0.56). NIR, near-infrared; MRI, magnetic resonance imaging; SBR, signal–background ratio; K*^trans^*, volume transfer constant; T1BR, ratio of gadolinium-enhanced T1 tumor signal to the brain.

The mechanism of Gd-MRI can only be influenced by vascular permeability (Montagne et al., 2016). Thus, we calculated the parameters K*^trans^*, K*^ep^*, V*^e^*, and V*^p^* using DCE-MRI to assess the relationship between permeability and NIR fluorescence. The SBR of NIR fluorescence was significantly positively correlated with K*^trans^* (*p* < 0.0001, R^2^ = 0.76) (Figure 5 and Table 2) but not K*^ep^* (*p* = 0.64), V*^e^* (*p* = 0.30), and V*^p^* (*p* = 0.32) (Table 2). Figure 6 shows a significant correlation between T1BR on MRI and K*^trans^* (*p* = 0.0013, R^2^ = 0.56). The linear regression plot revealed no significant association of T1BR with K*^ep^* (*p* = 0.70), V*^e^* (*p* = 0.96), or V*^p^* (*p* = 0.44) (Table 2).

### Near-Infrared Contrast Dye to Detect Margins

Finally, we tried to confirm whether ICG-NIR could detect the tumor and the dural tail by pathological examination. In 12 out of 15 cases, we collected 25 specimens of the surrounding dura mater that was the edge of the tumor attachment and the dural tail on preoperative MRI. We excluded three cases of inverted SBR because the surrounding dura mater showed lower fluorescence than the normal brain parenchyma. Then these specimens were pathologically diagnosed without knowing the results of the NIR fluorescence (Table 3). By using the NIR camera, 21 of 25 specimens were positive, and four were negative. In margin specimens, 17 were pathologically positive, and eight were negative. The ICG-NIR fluorescence system had a sensitivity of 94% [95% confidence interval (CI), 84.2–98.9], but its specificity decreased to 38% (95% CI, 16.4–47.6) (Table 3). The PPV was 76% (95% CI, 68.2–80.1), and the NPV was 75% (95% CI 32.9–95.3) (Table 3).

**TABLE 3:** Dura mater samples from the dural tail on pre-operative MRI in 25 samples from 12 patients.

NIR fluorescence				Sensitivity (95%CI)	Specificity (95%CI)	PPV (95%CI)	NPV (95%CI)
Tumor pathology		Positive	Negative				
	Positive	16	1	94% (84.2–98.0)	38% (16.4–47.6)	76% (68.2–80.1)	75% (32.9–95.3)
	Negative	5	3				

*Sensitivity: 94%, Specificity: 38%, Positive predictive value: 76%, Negative predictive value: 75%.*

*MRI, magnetic resonance imaging; NIR, near-infrared; PPV, positive predictive value; NPV, negative predictive value; CI, confidence interval.*

## Discussion

### Indocyanine Green Dose and Near-Infrared Fluorescence Intensity

Indocyanine green combines with albumin and is metabolized in the liver. Its biological halflife (t1/2) in blood and normal tissue is 3–4 min (De Gasperi et al., 2016). Prior to vascular surgery, 25 mg of ICG was intravenously administered to visualize the arterial, capillary, and venous flow. ICG has also been used for patients with brain tumors in the same manner (Ferroli et al., 2011; Kim et al., 2011); however, its use was not sufficient.

Previous studies of NIR using SWIG on tumors have affirmed its feasibility and usefulness. SWIG protocols described by Madajewski et al. (Madajewski et al., 2012) and Lee et al. (Lee et al., 2016, 2017a,b) include the administration of 5.0 mg/kg of ICG 24 h before surgery. We referred to a prior SWIG protocol (Hide et al., 2015) for several brain tumors carried out *via* a VisionSense exoscope and microscope (Lee et al., 2017b; Muto et al., 2021). However, Zeh et al. (2017) reported that the NIR-SBR in gliomas peaked at 1 h post-infusion, not 24 h. Considering these findings, we hypothesized that the administration of ICG more than 1 h before surgery might be enough and feasible for visualizing the tumor.

To verify our hypothesis, we intraoperatively administered 0.5 mg/kg of ICG with observation at ≥1 h post-injection in this study (DWIG). The mean time from injection to observation was 2.1 ± 0.9 h. We could observe fluorescence from the tumor after the intensity of the fluorescence decreased in the vascular structures and normal brain parenchyma. Delayed excretion occurred inside the tumor and may have been caused by enhanced permeability and retention (EPR) effects. EPR likely intensifies the difference in fluorescence intensity between the tumor and normal parenchyma. This study showed that DWIG was sufficient in differentiating NIR fluorescence in meningioma compared with normal brain parenchyma. In fact, the mean SBR of the intracranial meningioma in DWIG was 3.3 ± 2.6 compared with 5.6 ± 1.7 in SBR in SWIG (Lee et al., 2017a). The SBR in DWIG was slightly weaker than the SBR in SWIG in meningioma visualization but enough for differentiation between the tumor and normal brain parenchyma. This may improve the boost excitation of NIR (Li et al., 2019). Moreover, a lower dose of ICG in DWIG resulted in fewer effects. In the 15 cases in this study, there were no hematological and clinical side effects due to ICG usage. Thus, our technique could be useful for intraoperative real-time imaging in different populations including adolescents, young and older patients, patients with hepatic or renal dysfunction, and patients with other complications. Our findings suggested that NIR with microscopy is feasible for identifying intracranial meningiomas and a dural tail, although optimizing the settings is necessary to improve the visualization of tumor fluorescence. The procedure for DWIG is easier than that for SWIG, with a lower potential for side effects.

### Fluorophores in Intracranial Meningioma Resection

Neuronavigation is now popular for intracranial tumor removal. It facilitates surgical planning before surgery; however, the brain is displaced after opening the dura mater and during tumor removal. This decreases the accuracy of the tumor location. In fact, the fluorescence of ICG revealed unexpected lesions in the bright light field in a recurrent case after radiation therapy (Case 4). Thus, the NIR fluorescence technique would be beneficial as an adjunct for tumor removal.

Previous studies have reported that fluorescein, 5-ALA, and ICG are currently available as fluorescent agents, and fluorescein and 5-ALA are used for meningioma detection.

Indocyanine green has been used as a vascular angiography technique in patients with meningioma (Ueba et al., 2013). To date, 25 mg of ICG was intravenously administered to visualize the arterial, capillary, and venous flow. Nonetheless, SWIG with ICG has been used in recent years. Lee et al. (2017a) reported using the SWIG technique for meningioma with a VisionSense exoscope. SWIG involves the administration of 5 mg/kg of ICG 24 h before surgery. The maximum safe dosage of ICG for humans (Raabe et al., 2005) is 5 mg/kg. The usage of this maximum dosage in SWIG results in a metabolic burden on the liver and kidneys. To avoid side effects, such as liver and kidney dysfunction, patients younger than 18 years and pregnant women should not be considered for SWIG. In DWIG, these side effects are not a concern. ICG works by passive accumulation within tumor tissues *via* a delayed EPR effect. The benefits of fluorescence guidance with ICG are the negligible tissue autofluorescence in the NIR optical region, the low cost, and the long history of use in the neurosurgery field for monitoring blood flow during surgery. The drawbacks of ICG are that it is not a receptor-specific fluorophore, it has non-specific liver uptake, and it cannot be used in patients with iodine allergy, liver disease, or uremia. Lee and colleagues also demonstrated a sensitivity of 96.4%, specificity of 38.9%, PPV of 71.1%, and NPV of 87.5% (Lee et al., 2017a). The current study of DWIG showed a sensitivity of 94%, but its specificity decreased to 38%. The PPV and NPV were 76 and 75%, respectively, in intracranial meningiomas using a microscope, indicating that results are almost the same as SWIG. This pilot study confirmed the usefulness of DWIG in intracranial meningiomas using a microscope.

5-ALA is a cellular metabolic fluorophore in the heme biosynthesis pathway. 5-ALA-induced fluorescence is also influenced by the vascularity of the tumor, blood–brain barrier (BBB) permeability, tumor cell proliferative activity, and cellular density (Leon et al., 1996; Stummer et al., 1998). Although sensitivity and specificity rates in 5-ALA were inconsistent between studies, 5-ALA helps to visualize intracranial meningiomas with a sensitivity of 92–98% and a specificity of 95% (Dijkstra et al., 2019). There is no correlation between fluorescence intensity and WHO grade or histological subtype, and fluorescence is often heterogenic (Motekallemi et al., 2015; Foster and Eljamel, 2016). The drawbacks are phototoxicity and higher costs. 5-ALA fluorescence in different parts of the same tumor can present a different fluorescent pattern. Additionally, in dural imaging, 20% of patients with histologically confirmed meningioma invasion showed fluorescence in the dural tail (Wilbers et al., 2014). Most publications report cases or case series evaluating fluorescence status in intracranial meningiomas. Thus, the use of 5-ALA remains experimental, especially in cases of tumor recurrence, and it seems difficult to draw conclusions regarding the role of 5-ALA in meningioma surgery (Motekallemi et al., 2015; Foster and Eljamel, 2016).

Fluorescein sodium has been commonly used in identifying and monitoring intracranial angiography. Fluorescein sodium is able to penetrate the tumor because of the impaired BBB and tends not to diffuse into the normal brain. Although it can extend beyond Gd contrast-enhanced regions probably because of its smaller molecular weight, it corresponds to Gd uptake on MRI (Neira et al., 2017). Its merit is colorimetric separation, and the drawbacks are rapid photobleaching and non-specific high background. It has been safely used in humans for many years, predominantly in ophthalmology for retinal angiography, and the cost of fluorescein sodium is relatively low when compared with the cost of 5-ALA (Acerbi et al., 2014). Fluorescein sodium is usually visible to the naked eye at high dosages (20 mg/kg body weight) and is observable through the yellow 560-nm filter at lower doses, allowing better tissue discrimination with more natural colors (Schwake et al., 2015). Fluorescein sodium, like ICG, rapidly accumulates within tumor regions; however, the fluorescence may persist for hours aiding in the visualization of meningioma (Hadjipanayis, 2019). Fluorescein sodium has also been used in fluorescence-guided neurosurgery of glioblastoma and metastatic brain tumors (Okuda et al., 2010); however, the usefulness of fluorescein sodium is still controversial in intracranial meningioma surgery. In 30 patients, 88% of meningiomas showed homogenous tumor fluorescence, and 12% were heterogenous under a yellow, 560-nm wavelength filter (Akcakaya et al., 2017). Only three reports have been published about the experimental use of fluorescein sodium in meningioma surgery (da Silva et al., 2014a,b; Akcakaya et al., 2017).

Other fluorophores that can be directly targeted to specific receptors have been developed. BLZ-100 is a conjugate of the tumor-specific peptide chlorotoxin paired with an NIR fluorophore for use with recurrent glioblastoma and pediatric brain tumor (Patil et al., 2019). Alkylphosphocholine analogs are small synthetic phospholipid ether molecules, which may target specific tumor types including osteosarcoma, pancreatic adenocarcinoma, and glioblastoma (Weichert et al., 2014).

Epidermal growth factor receptor (EGFR) may be conjugated with fluorescent dyes, allowing for targeted cell-surface fluorescence (Seekell et al., 2013). While no clinical studies have been performed to date, preclinical studies have intravenously administered fluorescently conjugated anti-EGFR antibodies into rats and observed safe doses up to 24.5 mg/kg of an IRDye 800CW anti-EGFR antibody (ABY-029, Affibody, Sweden) (Samkoe et al., 2017).

Sprayable activatable fluorescent probes for glioblastoma have been developed. The fluorescent probe proline–arginine–HMRG reacts with calpain-1, which is highly expressed in glioblastoma cell line U87, and can distinguish tumors from peritumoral tissues *in vitro* (Hu et al., 2018). These fluorophores are still in the experimental stage and have been developed to mainly target glioblastoma, not meningioma. Hence, the fluorophores for meningioma are currently limited. Therefore, ICG-induced fluorescence enables feasible meningioma identification during surgery.

### Mechanism of Indocyanine Green Fluorescence

We hypothesized that ICG retention in diseased tissue was caused by the EPR effect, which, in turn, disturbs the lymphatic drainage system and increases vessel permeability through defective endothelial cells with wide fenestrations caused by an increase in agents from tumor cells (Maeda et al., 2016). ICG administration could be feasible for the optical monitoring of BBB disruption (Ergin et al., 2012). DCE-MRI can be used to evaluate BBB permeability and fluid and molecular exchange between the interstitial extracellular and intracellular fluid matrix (Tofts et al., 1999). In the dura mater, the BBB does not exist; thus, the mechanism of Gd and ICG retention in the dura mater seems to be different from that in brain tumors. The K*^trans^* score showed permeability from the intravascular to the extravascular space. We analyzed K*^trans^*, K*^ep^*, V*^e^*, V*^p^*, and cerebral blood volume on DCE-MRI. The mechanism of each parameter is illustrated in Figure 1. Only K*^trans^* showed a significant difference between the SBR and T1BR, but K*^ep^*, V*^e^*, and V*^p^* were not significantly different. Thus, only BBB permeability is correlated with ICG and Gd retention (Montagne et al., 2016). An improved DCE-MRI method using Gd-based contrast agents with an individual arterial input function curve has been developed to measure the BBB permeability constant K*^trans^* in the healthy brain as K*^trans^* values in a healthy brain are typically 10 to 100 times lower than those in patients with brain tumors, stroke, or infections. In our study, all patients with K*^trans^* > 0.1 showed visible fluorescence from ICG. Thus, K*^trans^* > 0.1 may be the threshold for the fluorescent visualization of intracranial meningioma.

### Ratio of T1–Gadolinium Enhancement to Normal Brain Ratio and Signal-to-Background Ratio

Some publications reported that T1BR is associated with the intensity of NIR fluorescence (Lee et al., 2017a; Muto et al., 2021). Our data demonstrated a correlation between T1BR and SBR and no correlation between T1BR and the maximum tumor diameter and tumor volume. These results suggest that T1BR might predict the efficacy of DWIG in tumor visualization before surgery. Additionally, the mechanism of Gd-MRI in brain tumors can only be influenced by vascular permeability (Montagne et al., 2016). This supports the hypothesis that retention of ICG occurs through the EPR effect in areas of enhanced vascular permeability (Maeda et al., 2000; Ergin et al., 2012). Accumulating data imply that BBB permeability is correlated with the fluorescent intensity in DWIG.

### Inverse Signal-to-Background Ratio

Three patients demonstrated an inverse pattern of NIR fluorescence in the current study. Lee et al. (2017a) previously reported this phenomenon and found no predictive factor for it. We observed significant differences between inversion and K*^trans^* and no difference in K*^ep^*, V*^e^*, V*^p^*, maximum diameter, tumor volume, T1BR, location, and WHO grade. K*^trans^*, indicating BBB permeability, may predict the inversion pattern, as well as the feasibility of SBR in intracranial meningiomas.

### Limitations of Delayed-Window Indocyanine Green

Our current study is a pilot study; therefore, the study sample was small. The sensitivity and specificity tests are largely determined by sample size, and thus, accurate test calculation was limited. Tumor location and surgical manipulations also might influence visualization by ICG. Because the concepts of SWIG and DWIG are new, a comparison study has not yet been performed. ICG is neither a receptor-bound nor a receptor-specific agent. Therefore, a lack of specificity is a concern for this method. The KINEVO system has an automatic exposure feature that can average the pixel intensity to normalize the background and assign it a neutral gray color. The camera tries to balance the exposure when set to the automatic exposure mode. To resolve these problems, we fixed the gain and illumination manually at the tumor view, resulting in the avoidance of false-positive tumor margins. Furthermore, the scores of these parameters can be used throughout the surgery. However, the scores are relative and not absolute values. Thus, the distance between the lens and the target was different each time when observing NIR fluorescence.

## Conclusion

This pilot study revealed the usefulness of the DWIG technique in identifying intracranial meningiomas and the dural tail in real time during surgery. NIR fluorescence of ICG can, on average, provide 3.3 ± 2.6 times stronger fluorescence in the meningioma than in normal brain parenchyma compared with 5.6 ± 1.7 in SWIG as reported previously. Gd-enhanced T1 tumor signals may predict NIR fluorescence of the intracranial meningioma. The BBB permeability as shown by K*^trans^* on DCE-MRI can contribute to Gd enhancement on MRI and to ICG retention and tumor fluorescence by NIR. This pilot study established the protocol for the main study. Further evaluation is needed to solve limitations for clinical use; however, we believe that the procedure for DWIG can be easier than that for SWIG, with a lower potential for side effects.

## Data Availability Statement

The original contributions presented in the study are included in the article/Supplementary Material, further inquiries can be directed to the corresponding author.

## Ethics Statement

The studies involving human participants were reviewed and approved by Fujita Health University Clinical Research Ethical Committee (CRB4180003) and Saiseikai Yokohamashi Tobu Hospital (20200137). The patients/participants provided their written informed consent to participate in this study.

## Author Contributions

JM, YM, KM, SY, DK, and MoH: editing and drafting the manuscript and abstract. JM, YM, MiH, JL, and YH: critically revising the work and reviewed the submitted version of the work. YH: supervision. JM and YM: conception and design. JM: approved the final version of the work on behalf of all authors.

## Conflict of Interest

JL owned stock options in Visionsense. The remaining authors declare that the research was conducted in the absence of any commercial or financial relationships that could be construed as a potential conflict of interest.

## Publisher’s Note

All claims expressed in this article are solely those of the authors and do not necessarily represent those of their affiliated organizations, or those of the publisher, the editors and the reviewers. Any product that may be evaluated in this article, or claim that may be made by its manufacturer, is not guaranteed or endorsed by the publisher.

## Supplementary Material

Supplementary Video 1 The movie of Case 1 shows the exact tumor location under bright light and near-infrared (NIR). At the end of surgery, we confirmed that the entire tumor was removed as no fluorescence was emitted from the tumor under bright light and NIR. NIR illumination can help surgeons to find lesions that are difficult to identify under bright light, especially in recurrent and radiated cases.

Supplementary Video 2 The movie of Case 2 demonstrates the tumor under bright light and near-infrared (NIR). At the end of surgery, we confirmed total tumor removal under bright light and NIR, and no fluorescence from the tumor and the dura mater remained.
